# Geriatric syndromes and intrinsic capacity in older adults with coronary heart disease: a cross-sectional study in China

**DOI:** 10.3389/fpubh.2026.1853890

**Published:** 2026-08-14

**Authors:** Jiaojiao Qiu, Shuaishuai Zhang, Yue Chen, Yan Wang, Yue Zhang, Nan Zhang, Qinzhi Cao, Guanchao Sun, Lin Zhou, Jin Sun, Man Li, Boyang Zeng, Zimei Chi, Shuxia Wang, Ping Zhu

**Affiliations:** 1 Department of Geriatrics, The Second Medical Center and National Clinical Research Center for Geriatric Diseases, Chinese PLA General Hospital, Beijing, China; 2 Medical School of Chinese PLA, Beijing, China

**Keywords:** coronary heart disease, geriatric syndromes, integrated care, intrinsic capacity, older adults

## Abstract

**Background:**

Geriatric syndromes (GS) are highly prevalent among community-dwelling older adults with self-reported physician-diagnosed coronary heart disease (CHD), but their association with intrinsic capacity (IC) remains poorly characterized.

**Methods:**

We analyzed cross-sectional data from 5,835 community-dwelling Chinese older adults with self-reported physician-diagnosed CHD (mean age 74.4 years; 65.8% women). GS burden (0 to ≥4) and specific comorbidities were examined against overall IC impairment and its five domains (locomotion, cognition, sensory, vitality, psychological). Adjusted prevalence ratios (PR) were estimated using multivariable modified Poisson regression models. Sensitivity analyses accounted for frailty, disability, and conceptual overlap between exposure and outcome domains, and the exclusion of severe end-stage deficits.

**Results:**

Overall, 69.6% of participants had ≥1 GS. IC impairment prevalence increased progressively with GS burden, from 35.6% (0 GS) to 84.7% (≥4 GS). Compared to those with 0 GS, participants with ≥4 GS had a substantially higher prevalence of overall IC impairment (PR = 2.00; 95% CI: 1.76–2.27), locomotion difficulties (PR = 6.52), and psychological problems (PR = 6.68; all *p* < 0.001). Individually, delirium symptoms showed the strongest association with cognitive (PR = 4.09) and overall IC impairment (PR = 1.48). Specific dyadic GS combinations (e.g., anxiety paired with chronic constipation) remained independently associated with IC impairment even after adjusting for the total concurrent GS burden. Sensitivity analyses confirmed that cumulative GS associations remained stable after adjusting for physical vulnerability (≥4 GS PR = 1.73) and excluding severe deficits. No significant interactions were observed by age, sex, or region.

**Conclusion:**

In community-dwelling older adults with self-reported physician-diagnosed CHD, a higher GS burden is strongly and independently associated with IC impairment, reflecting a functional continuum from early capacity declines to advanced physical vulnerability. This graded relationship persists even when accounting for physical vulnerability, highlighting the critical value of identifying high-risk GS clusters to proactively manage IC.

## Introduction

Coronary heart disease (CHD) is a leading cause of morbidity and mortality among older adults worldwide (1, 2). While advances in cardiovascular care have improved survival, many older adults with CHD experience significant multidomain physiological declines that cannot be fully explained by cardiac pathology alone. The rationale for emphasizing CHD in this context is critical. CHD is not merely an isolated localized organ pathology; rather, it acts as a systemic accelerator of biological aging. These individuals frequently exhibit chronic systemic inflammation, endothelial dysfunction, and impaired hemodynamics, which collectively diminish physiological reserves across the heart-brain axes (3–5). Consequently, individuals with CHD represent a highly vulnerable population wherein systemic inflammation driven by cardiovascular pathology accelerates biological aging (6) and synergizes with age-related deficits, making them exceptionally susceptible to rapid IC impairment. Growing evidence suggests that age-related conditions, collectively termed geriatric syndromes (GS), such as falls, cognitive impairment, and psychological distress, play a critical role in contributing to adverse outcomes in this population (7–10).

To holistically capture these complex physiological and capacity declines, the World Health Organization (WHO) introduced the framework of intrinsic capacity (IC), defined as the composite of all physical and mental capacities that an individual can draw upon at any point in time (11). Encompassing five core domains—locomotion, cognition, sensory function, vitality, and psychological status—the IC framework represents a vital paradigm shift in modern geriatrics. It transitions clinical focus from a traditional, reactive, disease-centric model to a proactive, function-oriented, and person-centered approach. Conceptually, it is crucial to distinguish the IC framework from the traditional frailty and GS models. While the frailty/GS paradigm is primarily a deficit-based approach built around identifying accumulated clinical limitations, physiological deficits, and later-stage vulnerability, IC represents a capacity-based approach that characterizes the totality of a person's preserved physical and mental capacities, enabling the identification of adverse aging trajectories often at earlier stages. However, it must be acknowledged that at the lower end of the functional continuum, severe capacity declines phenotypically converge with clinical frailty. In clinical practice, these progressive capacity declines (IC impairment) and advanced clinical deficits (GS) frequently intersect and coexist in highly vulnerable populations, such as older adults with CHD.

Evaluating IC provides a more precise and comprehensive lens to assess an older adult's physiological reserve, resilience, and true biological age than simply counting multimorbidity diagnoses. Impairment in IC is strongly predictive of disability, hospitalization, and mortality, independent of specific disease states (12–14). However, the relationship between the cumulative burden of deficit-based GS and the erosion of capacity-based IC in older adults with CHD remains poorly characterized. Most prior studies have examined GS or IC components in isolation, often overlooking their co-occurrence patterns and synergistic effects.

Emerging data suggest that certain GS, such as delirium symptoms, anxiety, and chronic constipation, may cluster together and disproportionately contribute to disability (15, 16). However, whether these specific dyadic or higher-order combinations are independently associated with a markedly higher prevalence of IC impairment in community-dwelling older adults with self-reported physician-diagnosed CHD remains unknown. Furthermore, it is unclear whether the association between GS burden and IC is consistent across demographic subgroups.

Using a large-scale, multicenter sample of older Chinese adults with CHD recruited from 14 provinces, this study aimed to quantify the prevalence of IC impairment across increasing GS burden and identify specific GS combinations associated with IC impairment. Additionally, we assessed the independent associations between individual GS and specific IC domains to provide a comprehensive understanding of these relationships. Our findings may inform integrated care strategies that address modifiable syndromes to support IC in older adults with cardiovascular disease.

## Methods

### Study design and participants

This analysis used data from the Comprehensive Longevity and Aging Cohort Study in China (CLASIC, ChiCTR2100047134), a nationwide, population-based cohort study of community-dwelling older adults conducted between 2023 and 2024. A multi-stage stratified sampling approach was employed. Specifically, 14 provinces or municipalities were initially selected to represent five major regions of China: Northwest, North, Central, Southwest, and East. Within each selected province, approximately one-third of its cities were randomly chosen, for a total of 69 cities. At the final stage, 4 to 6 community-level survey sites were identified per city, resulting in 352 survey locations distributed across the country. The enrolled population was aged ≥60 years. The baseline characteristics of the study participants are formally presented in Table 1. The study was approved by the Ethics Committee of the PLA General Hospital (approval number: S2021-134-01), and all participants provided written or electronic informed consent prior to data collection.

**Table 1:** Baseline characteristics of participants with and without GS among community-dwelling older adults with self-reported physician-diagnosed CHD.

Characteristics	Overall	Without GS	With GS	*p*-value
	(n = 5,835)	(n = 1,776)	(n = 4,059)	
Sex				
Female	3,839 (65.8)	1,093 (61.5)	2,746 (67.7)	< 0.001
Male	1,996 (34.2)	683 (38.5)	1,313 (32.3)	
Age (years), mean (SD)	74.38 (6.15)	73.80 (6.26)	74.63 (6.09)	< 0.001
60–64	279 (4.8)	113 (6.4)	166 (4.1)	< 0.001
65–69	1,044 (17.9)	360 (20.3)	684 (16.9)	
70–74	1,730 (29.6)	528 (29.7)	1,202 (29.6)	
75–79	1,539 (26.4)	442 (24.9)	1,097 (27.0)	
80–84	926 (15.9)	237 (13.3)	689 (17.0)	
≥85	317 (5.4)	96 (5.4)	221 (5.4)	
Ethnicity				
Han	5,792 (99.3)	1,761 (99.2)	4,031 (99.3)	0.639
Other	43 (0.7)	15 (0.8)	28 (0.7)	
National region				
Central China	602 (10.3)	103 (5.8)	499 (12.3)	< 0.001
Southwest China	1,308 (22.4)	341 (19.2)	967 (23.8)	
Northwest China	977 (16.7)	299 (16.8)	678 (16.7)	
East China	1,632 (28.0)	497 (28.0)	1,135 (28.0)	
North China	1,316 (22.6)	536 (30.2)	780 (19.2)	
Residence				
Rural	1,231 (21.1)	466 (26.2)	765 (18.8)	< 0.001
Urban	4,604 (78.9)	1,310 (73.8)	3,294 (81.2)	
Education level				
College and higher	732 (12.5)	235 (13.2)	497 (12.2)	< 0.001
Middle and high school	3,475 (59.6)	1,128 (63.5)	2,347 (57.8)	
Primary school or less	1,628 (27.9)	413 (23.3)	1,215 (29.9)	
Marital status				
Married	4,628 (79.3)	1,479 (83.3)	3,149 (77.6)	< 0.001
Widowed	1,092 (18.7)	274 (15.4)	818 (20.2)	
Divorced, separated, or never married	115 (2.0)	23 (1.3)	92 (2.3)	
Dwelling status				
Non-solitude	4,761 (81.6)	1,489 (83.8)	3,272 (80.6)	0.004
Solitude	1,074 (18.4)	287 (16.2)	787 (19.4)	
Occupation				
Unemployed	201 (3.4)	47 (2.6)	154 (3.8)	0.057
Manual	3,859 (66.1)	1,207 (68.0)	2,652 (65.3)	
Trades	361 (6.2)	97 (5.5)	264 (6.5)	
Cadre	604 (10.4)	172 (9.7)	432 (10.6)	
Professional	698 (12.0)	213 (12.0)	485 (11.9)	
Other	112 (1.9)	40 (2.3)	72 (1.8)	
Alcohol consumption				
No	4,956 (84.9)	1,547 (87.1)	3,409 (84.0)	0.006
Ever	234 (4.0)	56 (3.2)	178 (4.4)	
Now	645 (11.1)	173 (9.7)	472 (11.6)	
Smoking status				
Never smoker	5,100 (87.4)	1,589 (89.5)	3,511 (86.5)	0.005
Former smoker	356 (6.1)	95 (5.3)	261 (6.4)	
Current smoker	379 (6.5)	92 (5.2)	287 (7.1)	
Frailty (%)	2,347 (40.2)	421 (23.7)	1,926 (47.5)	< 0.001
Disability (%)	251 (4.3)	33 (1.9)	218 (5.4)	< 0.001
No. of chronic diseases, mean (SD)	3.17 (1.68)	2.58 (1.52)	3.43 (1.68)	< 0.001
IC score, mean (SD)	90.26 (12.35)	94.75 (8.01)	88.29 (13.37)	< 0.001
IC impairment (%)	3,156 (54.1)	669 (37.7)	2,487 (61.3)	< 0.001

Data are *n* (%) or mean (SD) unless specified otherwise. *T*-test was performed for continuous variables, and the χ^2^ test was conducted for categorical variables. GS, geriatric syndrome; CHD, coronary heart disease; IC, intrinsic capacity.

### CHD

The presence of CHD was determined via a standardized questionnaire administered by trained interviewers. Participants were asked the following specific question: “Has a doctor ever diagnosed you with any of the diseases listed below?” CHD was considered present if a participant reported a prior formal diagnosis of coronary heart disease by a physician.

### Geriatric syndromes

We assessed nine GS using validated, culturally adapted instruments developed for the Chinese population: falls (self-reported fall in the past year), high osteoporosis risk (One-Minute Osteoporosis Risk Test score ≥5) (17), dysphagia (EAT-10 score ≥3) (18), chronic constipation (Bristol Stool Form Scale types 1 or 2) (19), anxiety (SAS score ≥50) (20), delirium symptoms (defined by a CAM-CR score ≥20, which indicates high risk or subsyndromal symptoms rather than active clinical delirium) (21), chronic pain (assessed via FPS-R) (22), poor sleep quality (PSQI score >5) (23), and polypharmacy (daily use of five or more medications). The total GS burden was quantified by summing the number of present syndromes. For analysis, participants were categorized by GS count: 0 (reference) 1, 2, 3, or ≥4 GS.

### Intrinsic capacity

IC was assessed across five domains: cognition (Chinese Mini-Mental State Examination, MMSE) (24), psychological status (15-item Geriatric Depression Scale, GDS-15) (25), vitality (Mini-Nutritional Assessment Short-Form, MNA-SF) (26), sensory function (self-reported vision and hearing), and locomotion (assessed using two specific items on walking and stair-climbing difficulty derived from the SARC-F questionnaire) (27). To standardize the assessment across these heterogeneous instruments, raw scores or categorical responses from each domain were mathematically transformed into a common 0–20 scale, with 0 indicating severe impairment, 10 indicating mild to moderate impairment, and 20 representing normal function. Impairment within any single domain was defined as a transformed score of less than 20 (i.e., 10 or 0). The specific raw assessment criteria, transformation procedures, and cutoffs used to define impairment for each domain are detailed in Supplementary Table 1. The overall composite assessment of IC was operationalized based on the conceptual framework of the World Health Organization (WHO) Integrated Care for Older People (ICOPE) guidelines and established epidemiological methodologies for large-scale cohorts (28). Based on this framework, participants were categorized into three levels based on their total score (range 0–100): robust IC (score = 100), mildly impaired IC (80–99), or significantly reduced IC (less than 80). Overall IC impairment was defined as the presence of impairment in ≥1 domain.

### Covariates

Sociodemographic and health-related covariates included age, sex, ethnicity, marital status, living arrangement, education level, geographic region, occupation, smoking history, and alcohol consumption. Participants' residence was grouped into five administrative regions of China: Northwest, North, Central, Southwest, and East. Urban settings were defined as developed areas, while rural locales were classified as less developed. Smoking and alcohol use were categorized as: never used, current use (regular consumption within the past 6 months), or former use (prior use but none in the preceding 6 months). Physical health indicators were measured using standardized protocols, specifically including assessments of frailty and ADL disability. Frailty was assessed with the FRAIL scale, where 0 indicates robustness and ≥1 indicates prefrailty/frailty (29). ADL disability was defined as needing help with ≥1 ADL task based on the Barthel Index (30).

### Statistical analysis

Differences in continuous variables were evaluated using Student's t-test, while associations for categorical variables were tested with the chi-squared (χ^2^) test. Age-adjusted prevalence estimates were derived using the age distribution of the Chinese population in 2020 as the reference (31). Baseline characteristics and subgroup analyses were generated using the tableone (32) and jstable (33), while data visualization was performed utilizing ggplot2 (34). Because the outcome of interest (IC impairment) was common in this cohort (>10%), modified Poisson regression models with robust error variance were employed to compute adjusted prevalence ratios (PR) and 95% confidence intervals (CI) for the association between GS burden and IC impairment (35, 36) to avoid overestimating the effect size typically seen with odds ratios in cross-sectional designs (37). The primary model was adjusted for sociodemographic and clinical factors, including age, sex, ethnicity, national region, residence, education level, marital status, occupation, dwelling status, alcohol consumption, smoking status, and number of chronic diseases.

The analyses investigating specific co-occurring geriatric syndrome pairs were considered exploratory. To account for multiple comparisons, *p*-values for these pairwise associations were adjusted using the Benjamini–Hochberg False Discovery Rate (FDR) procedure. Furthermore, multivariable models assessing these specific pairs were additionally adjusted for the total geriatric syndrome burden (defined as the total count of concurrent syndromes minus the specific pair being evaluated) to isolate the combination's independent association from the cumulative burden effect. Sensitivity analyses were further performed by additionally adjusting for frailty and disability to assess the independence of the associations.

To address the potential for artificially inflated associations due to conceptual overlap between specific GS and IC domains (e.g., delirium symptoms and cognition; anxiety and psychological status; falls/pain and locomotion; dysphagia and vitality), an additional sensitivity analysis was conducted. In these models, we recalculated the total GS burden by explicitly excluding the specific overlapping syndromes before evaluating its association with the corresponding IC domain. Finally, to rigorously address the potential conceptual overlap between end-stage clinical deficits and intrinsic capacity decline, a third sensitivity analysis was conducted by explicitly excluding individuals with severe cognitive impairment (defined as MMSE < 18) and severe malnutrition (defined as MNA-SF ≤ 7).

Forest plots were created with the forestploter package (38). Subgroup analyses were conducted by stratifying the sample by sex, region, and age categories (< 75 years vs. ≥75 years). In these models, the group with 0 geriatric syndromes served as the reference group, and models were adjusted for all aforementioned covariates except the stratification variable. Interactions were tested by adding product terms to the models. All statistical analyses were conducted in RStudio (version 4.4.1; R Foundation for Statistical Computing, Vienna, Austria) (39), with statistical significance defined as a two-sided *p*-value < 0.05.

## Results

**Figure 1:**
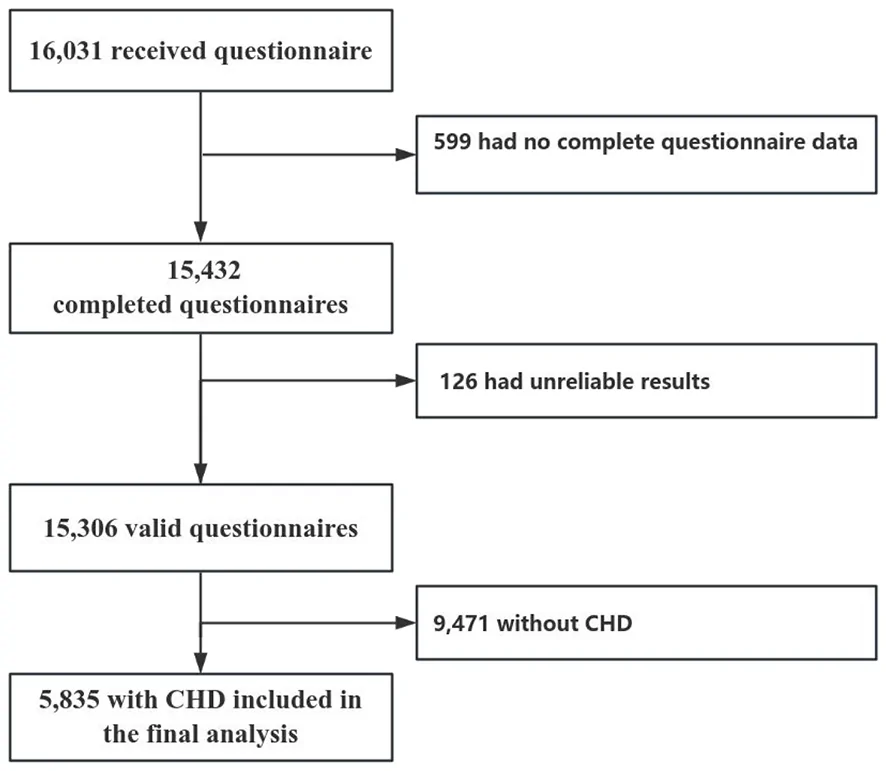
Study flowchart.

Participants were excluded from the study if they met any of the following criteria: (1) missing or incomplete questionnaire data (*n* = 599); (2) unreliable responses (defined as evident logical inconsistencies, *n* = 126); or (3) absence of CHD (*n* = 9,471). After excluding these individuals, 5,835 participants were included in the final analysis (Figure 1).

Among the 5,835 community-dwelling participants with self-reported physician-diagnosed CHD, 65.8% were female. The mean age was 74.38 ± 6.15 years, and the mean IC score was 90.26 ± 12.35. A total of 4,059 (69.6%) had at least one GS. Compared to those without GS, participants with GS were significantly older (74.63 ± 6.09 vs. 73.80 ± 6.26 years), and more likely to be female (67.7% vs. 61.5%). Furthermore, the GS group had a higher prevalence of frailty (47.5% vs. 23.7%), disability (5.4% vs. 1.9%), and IC impairment (61.3% vs. 37.7%), as well as a greater number of chronic conditions (3.43 ± 1.68 vs. 2.58 ± 1.52) (Table 1). Further detailed characteristics regarding the distribution of specific geriatric syndromes are provided in Supplementary Table 2. Notably, the prevalence of every individual GS was significantly higher among participants with IC impairment than in those with robust IC (all *p* < 0.001). This group also demonstrated a significantly heavier cumulative syndromic burden (*p* < 0.001).

**Figure 2:**
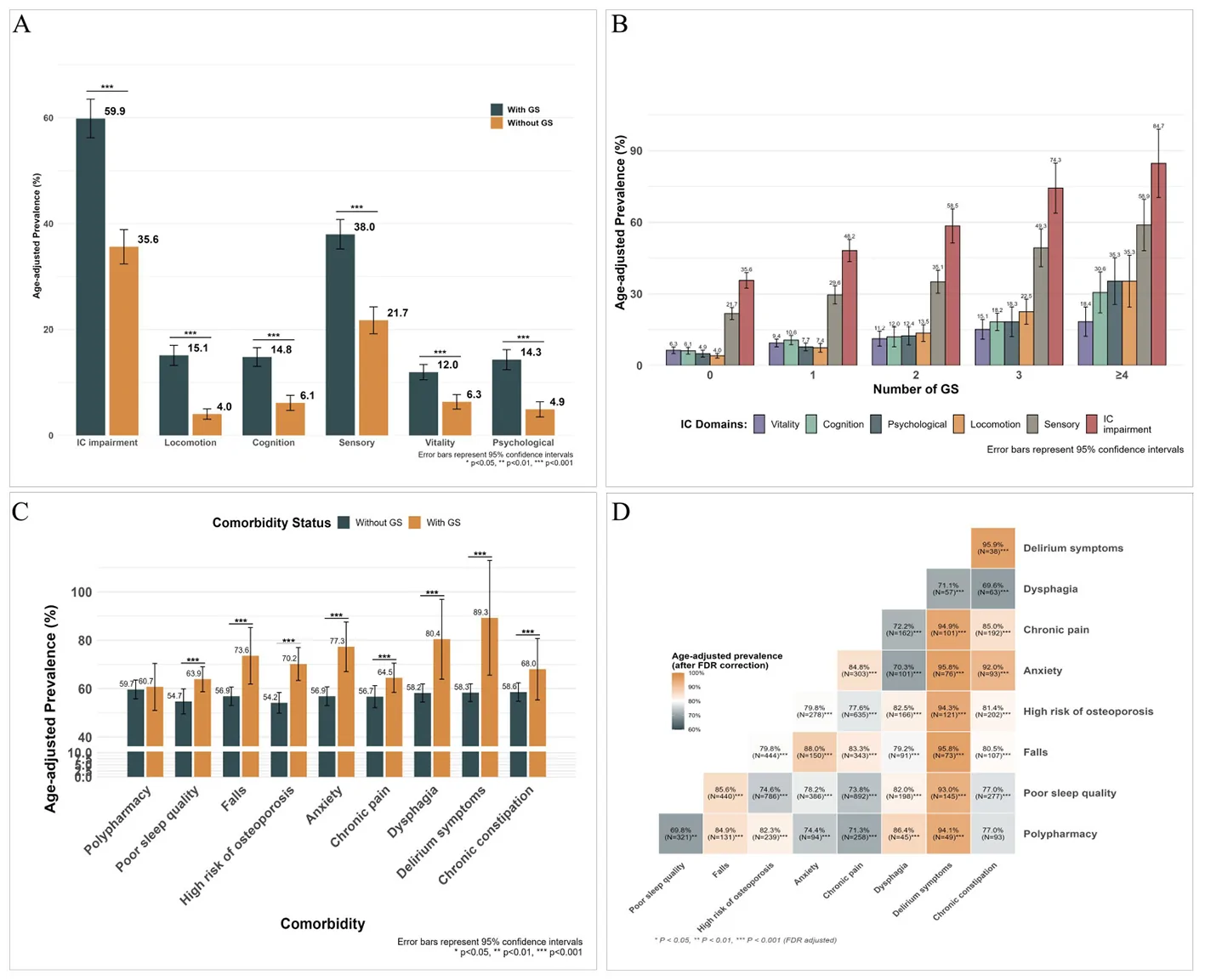
Age-adjusted prevalence of IC impairment across five domains among community-dwelling older adults with self-reported physician-diagnosed CHD, by **(A)** GS status, **(B)** number of GS, **(C)** individual GS conditions, and **(D)** dyadic GS comorbidity patterns. For dyadic patterns in panel D, *p*-values were adjusted for multiple comparisons using the Benjamini–Hochberg False Discovery Rate (FDR) procedure. The absolute number of participants (*N*) for each combination is presented within the figure.

The age-adjusted prevalence of IC impairment was markedly higher in participants with ≥1 GS than in those without (59.9% vs. 35.6%, *p* < 0.001), consistent with their lower mean IC scores (88.29 ± 13.37 vs. 94.75 ± 8.01). Across the five IC domains, sensory impairments were the most prevalent (38.0% vs. 21.7%), followed by locomotion difficulties (15.1% vs. 4.0%), cognitive impairment (14.8% vs. 6.1%), psychological problems (14.3% vs. 4.9%), and inadequate vitality (12.0% vs. 6.3%) (all *p* < 0.001) (Figure 2A).

The prevalence of all IC impairments increased progressively with GS burden (Figure 2B). Participants with ≥4 GS exhibited the highest rates of overall IC impairment (84.7%), sensory impairments (58.9%), and both locomotion and psychological problems (35.3%). In contrast, individuals completely free of GS (0 GS) demonstrated the lowest baseline prevalence across all matched domains (overall IC impairment: 35.6%; sensory: 21.7%; vitality: 6.3%; cognition: 6.1%; psychological: 4.9%; locomotion: 4.0%). This progressive escalation from 0 to ≥4 GS highlights a robust dose-response association between cumulative geriatric burden and IC impairment.

These highly prevalent comorbidities, including delirium symptoms and dysphagia, were closely associated with the severe IC impairment observed in the GS group (Figure 2C). Specifically, among participants burdened with GS, the prevalence of overall IC impairment was as high as 89.3% in those with delirium symptoms, 80.4% in those with dysphagia, and 77.3% in those with anxiety, all of which were significantly higher than the corresponding rates in the non-GS group (all *p* < 0.001). Regarding dyadic GS patterns among participants with CHD, the co-occurrence of delirium symptoms with any other GS (excluding dysphagia) was associated with remarkably high IC impairment rates, ranging from 94.1% to 95.9% (Figure 2D). Notably, the anxiety–chronic constipation pair also exhibited a high prevalence of IC impairment (92.0%), followed by the anxiety–falls combination (88.0%). These results suggest that specific clusters of comorbidities are particularly indicative of IC impairment in this population.

However, to determine whether the apparent impact of these specific high-risk pairs was simply driven by a higher overall number of syndromes, we performed sensitivity analyses additionally adjusting for the remaining cumulative GS burden (total GS count excluding the evaluated pair). While some combinations were attenuated, the co-occurrence of anxiety with chronic constipation (PR = 1.29, 95% CI: 1.04–1.61, *p* = 0.023) and poor sleep quality with delirium symptoms (PR = 1.24, 95% CI: 1.03–1.49, *p* = 0.021) remained independently and significantly associated with IC impairment (Supplementary Table 3).

Modified Poisson regression models confirmed a strong, graded association between GS burden and IC impairment (Table 2). Compared with those without GS, participants with ≥4 GS exhibited the highest risk across all dimensions, including overall IC impairment (PR = 2.00, 95% CI: 1.76–2.27), locomotion difficulties (PR = 6.52, 95% CI: 4.96–8.56), psychological problems (PR = 6.68, 95% CI: 5.11–8.74), cognitive impairment (PR = 3.85, 95% CI: 2.98–4.97), sensory impairments (PR = 2.24, 95% CI: 1.92–2.62), and inadequate vitality (PR = 2.47, 95% CI: 1.86–3.26; all *p* < 0.001).

**Table 2:** The association of GS burden and specific GS comorbidities with IC and five functional domains in community-dwelling older adults with self-reported physician-diagnosed CHD.

Variables	IC impairment	Locomotion	Cognition	Sensory	Vitality	Psychological
	PR (95% CI, *p*-value)	PR (95% CI, *p*-value)	PR (95% CI, *p*-value)	PR (95% CI, *p*-value)	PR (95% CI, *p*-value)	PR (95% CI, *p*-value)
No. of GS						
0	1.00 (reference)	1.00 (reference)	1.00 (reference)	1.00 (reference)	1.00 (reference)	1.00 (reference)
1	1.29 (1.16–1.43, *p* < 0.001)	1.69 (1.28–2.22, *p* < 0.001)	1.62 (1.29–2.05, *p* < 0.001)	1.29 (1.13–1.47, *p* < 0.001)	1.45 (1.15–1.83, *p* = 0.002)	1.52 (1.16–1.98, *p* = 0.002)
2	1.50 (1.34–1.67, *p* < 0.001)	2.82 (2.14–3.70, *p* < 0.001)	1.77 (1.37–2.28, *p* < 0.001)	1.48 (1.29–1.71, *p* < 0.001)	1.60 (1.24–2.06, *p* < 0.001)	2.35 (1.79–3.08, *p* < 0.001)
3	1.85 (1.64–2.10, *p* < 0.001)	4.54 (3.43–6.01, *p* < 0.001)	2.64 (2.02–3.45, *p* < 0.001)	2.01 (1.73–2.35, *p* < 0.001)	2.02 (1.52–2.67, *p* < 0.001)	3.44 (2.59–4.59, *p* < 0.001)
≥4	2.00 (1.76–2.27, *p* < 0.001)	6.52 (4.96–8.56, *p* < 0.001)	3.85 (2.98–4.97, *p* < 0.001)	2.24 (1.92–2.62, *p* < 0.001)	2.47 (1.86–3.26, *p* < 0.001)	6.68 (5.11–8.74, *p* < 0.001)
Comorbidity						
Polypharmacy						
No	1.00 (reference)	1.00 (reference)	1.00 (reference)	1.00 (reference)	1.00 (reference)	1.00 (reference)
Yes	1.06 (0.96–1.18, *p* = 0.244)	1.51 (1.26–1.82, *p* < 0.001)	1.30 (1.07–1.59, *p* = 0.009)	1.09 (0.96–1.24, *p* = 0.192)	0.97 (0.76–1.23, *p* = 0.777)	1.02 (0.81–1.28, *p* = 0.860)
Poor sleep quality						
No	1.00 (reference)	1.00 (reference)	1.00 (reference)	1.00 (reference)	1.00 (reference)	1.00 (reference)
Yes	1.31 (1.22–1.41, *p* < 0.001)	1.78 (1.53–2.07, *p* < 0.001)	1.56 (1.34–1.81, *p* < 0.001)	1.39 (1.27–1.52, *p* < 0.001)	1.36 (1.16–1.60, *p* < 0.001)	1.87 (1.59–2.19, *p* < 0.001)
Falls						
No	1.00 (reference)	1.00 (reference)	1.00 (reference)	1.00 (reference)	1.00 (reference)	1.00 (reference)
Yes	1.33 (1.21–1.46, *p* < 0.001)	2.75 (2.35–3.23, *p* < 0.001)	1.63 (1.37–1.95, *p* < 0.001)	1.34 (1.19–1.50, *p* < 0.001)	1.31 (1.07–1.61, *p* = 0.010)	2.15 (1.81–2.57, *p* < 0.001)
High risk of osteoporosis						
No	1.00 (reference)	1.00 (reference)	1.00 (reference)	1.00 (reference)	1.00 (reference)	1.00 (reference)
Yes	1.37 (1.27–1.49, *p* < 0.001)	2.09 (1.79–2.44, *p* < 0.001)	1.54 (1.31–1.80, *p* < 0.001)	1.46 (1.33–1.61, *p* < 0.001)	1.57 (1.32–1.87, *p* < 0.001)	2.41 (2.05–2.83, *p* < 0.001)
Anxiety						
No	1.00 (reference)	1.00 (reference)	1.00 (reference)	1.00 (reference)	1.00 (reference)	1.00 (reference)
Yes	1.39 (1.26–1.54, *p* < 0.001)	1.99 (1.65–2.39, *p* < 0.001)	1.94 (1.61–2.33, *p* < 0.001)	1.48 (1.30–1.67, *p* < 0.001)	1.63 (1.32–2.02, *p* < 0.001)	3.73 (3.15–4.42, *p* < 0.001)
Chronic pain						
No	1.00 (reference)	1.00 (reference)	1.00 (reference)	1.00 (reference)	1.00 (reference)	1.00 (reference)
Yes	1.21 (1.12–1.31, *p* < 0.001)	1.77 (1.52–2.06, *p* < 0.001)	1.35 (1.16–1.58, *p* < 0.001)	1.33 (1.21–1.46, *p* < 0.001)	1.06 (0.89–1.27, *p* = 0.506)	1.38 (1.17–1.63, *p* < 0.001)
Dysphagia						
No	1.00 (reference)	1.00 (reference)	1.00 (reference)	1.00 (reference)	1.00 (reference)	1.00 (reference)
Yes	1.40 (1.23–1.59, *p* < 0.001)	2.59 (2.10–3.18, *p* < 0.001)	2.07 (1.66–2.59, *p* < 0.001)	1.53 (1.32–1.79, *p* < 0.001)	2.13 (1.67–2.71, *p* < 0.001)	2.82 (2.28–3.49, *p* < 0.001)
Delirium symptoms						
No	1.00 (reference)	1.00 (reference)	1.00 (reference)	1.00 (reference)	1.00 (reference)	1.00 (reference)
Yes	1.48 (1.28–1.73, *p* < 0.001)	2.33 (1.82–2.99, *p* < 0.001)	4.09 (3.33–5.02, *p* < 0.001)	1.56 (1.30–1.87, *p* < 0.001)	1.87 (1.38–2.53, *p* < 0.001)	3.74 (2.98–4.69, *p* < 0.001)
Chronic constipation						
No	1.00 (reference)	1.00 (reference)	1.00 (reference)	1.00 (reference)	1.00 (reference)	1.00 (reference)
Yes	1.25 (1.12–1.39, *p* < 0.001)	1.79 (1.47–2.18, *p* < 0.001)	1.51 (1.23–1.87, *p* < 0.001)	1.20 (1.05–1.38, *p* = 0.008)	1.61 (1.29–2.01, *p* < 0.001)	1.69 (1.37–2.09, *p* < 0.001)

The model was adjusted for age, sex, ethnicity, national region, residence, education level, marital status, occupation, dwelling status, alcohol consumption, smoking status, and number of chronic diseases. The adjusted PR were estimated using multivariable modified Poisson regression models. PR, prevalence ratio; CI, confidence interval; IC, intrinsic capacity, GS, geriatric syndrome.

Among individual GS conditions, delirium symptoms exhibited the strongest domain-specific associations, particularly with cognitive impairment (PR = 4.09, 95% CI: 3.33–5.02), psychological problems (PR = 3.74, 95% CI: 2.98–4.69), and overall IC impairment (PR = 1.48, 95% CI: 1.28–1.73; all *p* < 0.001). Anxiety was also strongly associated with psychological problems (PR = 3.73, 95% CI: 3.15–4.42) and IC impairment (PR = 1.39, 95% CI: 1.26–1.54). Chronic constipation was consistently linked to impairment across all five IC domains (all *p* < 0.01). In contrast, polypharmacy showed more selective associations, primarily with locomotion difficulties (PR = 1.51, 95% CI: 1.26–1.82) and cognitive impairment (PR = 1.30, 95% CI: 1.07–1.59), while its association with overall IC impairment (PR = 1.06, 95% CI: 0.96–1.18) and other domains did not reach statistical significance.

**Figure 3:**
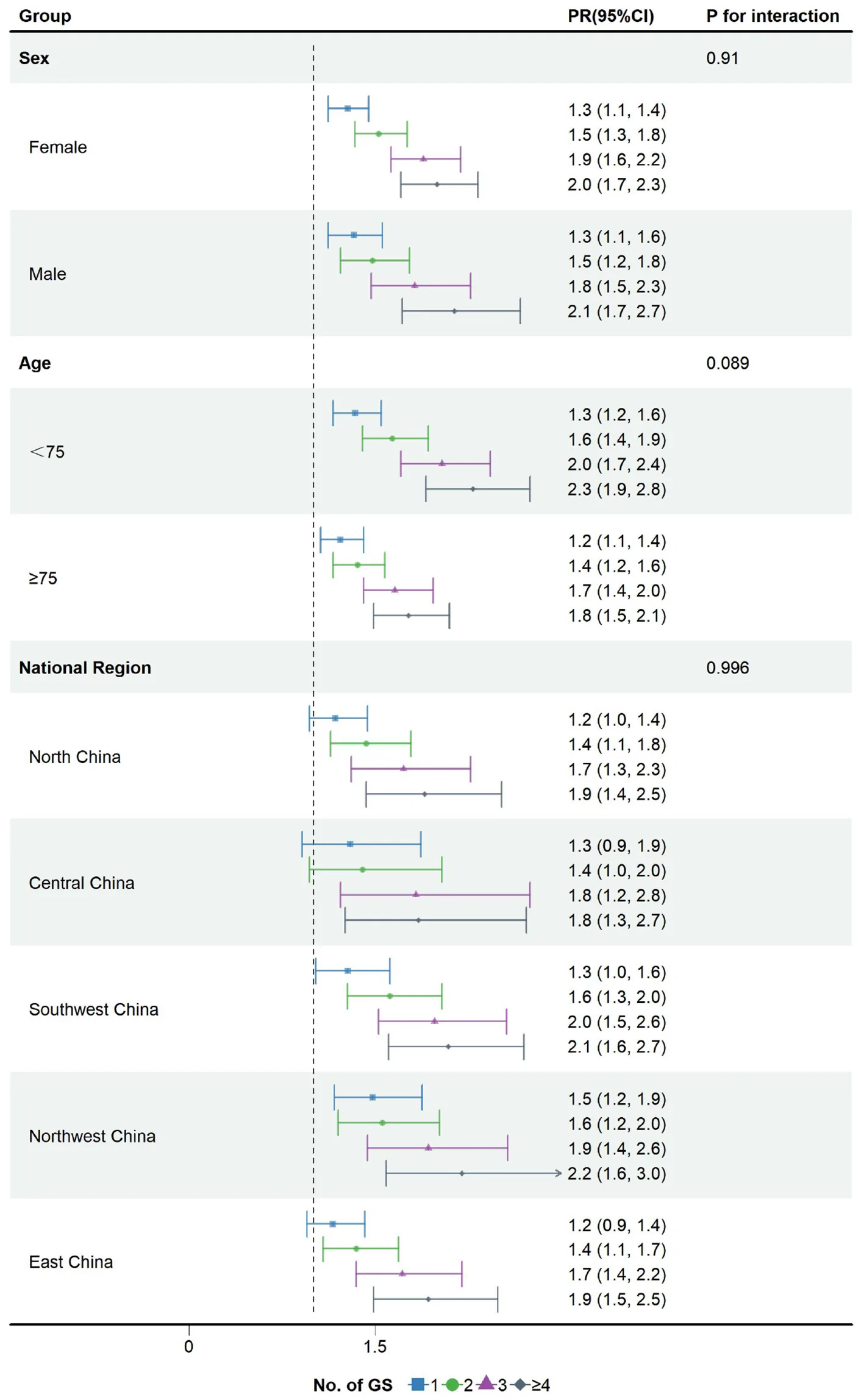
Forest plot of the association between number of GS and IC impairment across different subgroups. Subgroup analyses were stratified by sex, age categories (< 75 years and ≥75 years), and national region. Models were adjusted for all covariates included in the primary model (age, sex, ethnicity, national region, residence, education level, marital status, occupation, dwelling status, alcohol consumption, smoking status, and number of chronic diseases), excluding the specific stratification variable used in each subgroup analysis. The reference groups were individuals without any GS. PR, prevalence ratio; CI, confidence interval; IC, intrinsic capacity; GS, geriatric syndrome.

Subgroup analyses revealed no significant interactions between GS burden and sex (*p* = 0.91), age (*p* = 0.089), or region (*p* = 0.996) in relation to IC impairment (Figure 3). Across all examined subgroups, the prevalence of IC impairment rose consistently and significantly with an increasing GS count, demonstrating a graded association between cumulative GS and IC impairment. Sensitivity analysis further confirmed this association; after adjusting for frailty and disability, the ≥4 GS group maintained the highest risk for overall IC impairment (PR = 1.73, 95% CI: 1.52–1.97). This highlights that cumulative GS is strongly associated with IC, an association that persists even after adjusting for frailty and disability (Supplementary Table 4).

Furthermore, to address the potential conceptual overlap between end-stage geriatric deficits and early capacity decline, we performed an additional sensitivity analysis by explicitly excluding patients with severe cognitive impairment (MMSE < 18) and severe malnutrition (MNA-SF ≤ 7). Even within this mild-to-moderate cohort, the stepwise, independent association between cumulative GS burden and IC impairment remained robustly significant across overall IC and its five domains, with the ≥4 GS group exhibiting a 2.01-fold higher risk for overall IC impairment (PR = 2.01, 95% CI: 1.76–2.29; all *p* < 0.001) (Supplementary Table 5).

Sensitivity analyses for conceptual overlap: To ensure the observed associations were not artifacts of shared measurement constructs, we performed sensitivity analyses by excluding conceptually overlapping exposure-outcome pairs (e.g., excluding delirium symptoms when analyzing cognition; anxiety when analyzing psychological status; falls and pain when analyzing locomotion; and dysphagia when analyzing vitality). After re-calculating the total GS burden by excluding these specific syndromes, the associations between cumulative GS burden and the corresponding IC domains remained statistically significant (Supplementary Table 6), suggesting that our findings reflect true IC impairment rather than measurement overlap.

## Discussion

In this large-scale, multicenter study of older adults with CHD, we observed a strong and graded association between GS burden and IC impairment. Even a modest accumulation of two or more GS was linked to significantly higher prevalence of IC impairment, with prevalence escalating sharply as the syndrome count increased. Notably, specific dyadic combinations such as delirium symptoms paired with any other GS (excluding dysphagia) and the co-occurrence of anxiety and chronic constipation were associated with IC impairment rates exceeding 90%. Our initial analyses highlighted these specific GS pairs as having exceptionally high rates of IC impairment. Crucially, our subsequent sensitivity analyses revealed that while the overall cumulative GS burden is a primary factor associated with IC impairment, specific co-occurring conditions—such as anxiety paired with chronic constipation, or poor sleep quality with delirium symptoms—maintain an independent and statistically significant association with IC impairment. This suggests a dual pathway of physiological reserve depletion: the systemic accumulation of deficits broadly compromises IC, but specific high-risk syndromic clusters exert an additional, synergistic effect that further exacerbates this deterioration. Therefore, in clinical practice for older adults with CHD, a holistic assessment of the total syndromic burden must be integrated with targeted vigilance for these exceptionally high-risk combinations.

Our findings extend prior evidence by contextualizing GS within the WHO's IC framework, specifically among older adults with established CHD. While GS have long been recognized as indicators of adverse outcomes in aging populations (16), their cumulative association with overall IC in CHD has been underappreciated (40). CHD is often managed through a purely organ-specific approach, with insufficient attention to IC and frailty. Previous studies in general older adults cohorts reported an age-adjusted IC impairment prevalence ranging from 42.9 to 75.3% (41–43). In our study, the overall prevalence of IC impairment among individuals with CHD was 54.1%, which rose to 59.9% in those with ≥1 GS and reached 84.7% among those with four or more GS. The magnitude of association observed here exceeds estimates from general older adults cohorts, suggesting that the presence of CHD may amplify vulnerability to IC impairment when compounded by age-related syndromes. From a pathophysiological perspective, CHD and accelerated biological aging share common pathways, including chronic low-grade inflammation, oxidative stress, and endothelial dysfunction (3–6). Furthermore, chronic cardiac insufficiency and microvascular disease can lead to systemic hypoperfusion, which may directly compromise target organs such as the brain and skeletal muscle (44). This provides a biological basis for our findings, suggesting that the presence of CHD may amplify vulnerability to multidomain capacity declines when compounded by age-related syndromes.

This study reveals a clear and progressive association between the number of coexisting GS and IC impairment in older adults with CHD. As GS burden increased from zero to four or more syndromes, the risk of overall IC impairment rose significantly in a stepwise manner. This graded relationship suggests that GS do not occur in isolation, and their accumulation is associated with lower physiological reserves across multiple domains. Prior population-based research has shown that individuals with ≥3 GS faced a 6.6-fold risk of disability in activities of daily living compared to those without such conditions (45). The additive or synergistic interactions among these syndromes likely exacerbate frailty and capacity loss, underscoring the importance of viewing GS burden as a clinical marker of multisystem frailty rather than a collection of unrelated conditions (7).

Importantly, a conceptual and operational distinction must be made between IC and physical frailty. As highlighted by our methodological parameters, severe impairment across multiple IC domains in this specific high-burden CHD cohort phenotypically overlaps with clinical frailty. While IC and frailty approach aging from opposite perspectives—with IC focusing on the trajectory of preserved multidomain capacities and frailty focusing on the accumulation of later-stage deficits—they invariably converge at the lower end of the functional continuum. Therefore, the pronounced IC impairment observed in our participants with severe end-stage deficits should be interpreted as encompassing varying degrees of physical frailty.

Our multivariable analyses demonstrate a significant association between high GS burden and impairment across nearly all domains of IC in older adults with CHD. Specifically, anxiety was strongly associated with IC impairment (PR = 1.39, 95% CI: 1.26–1.54), and expectedly, showed the strongest association with psychological problems (PR = 3.73, 95% CI: 3.15–4.42) due to the inherent conceptual overlap between these domains. Beyond this expected overlap, the striking association with psychological morbidity is consistent with an expanding body of evidence supporting a bidirectional relationship between mental health and physical frailty in aging populations (46). Consistent with our findings, prior research has shown that greater social support and better physical functioning are associated with reduced anxiety and depression over time, while poorer psychosocial resources correlate with worse health-related quality of life outcomes (47). Importantly, sensory impairments, often seen as inevitable in aging, were significantly linked to higher GS burden. This challenges the view that sensory impairment is solely age-related and instead frames it as part of multisystem dysregulation, suggesting it may be modifiable even in advanced age. Basic science evidence shows that sensory perception actively regulates systemic physiology, with genetic studies demonstrating that sensory neurons influence energy balance, metabolism, and longevity via neuroendocrine pathways (48). In contrast, vitality exhibited the weakest association with GS burden among all IC domains in older adults with CHD, suggesting its resilience to acute or subacute syndromes and highlighting its distinct, socially and functionally rooted nature. This supports viewing vitality as a multidimensional construct requiring composite assessment (49). The observed heterogeneity across IC domains highlights that capacity impairment is not uniform but rather unfolds along divergent pathways shaped by underlying biological, clinical, and psychosocial factors.

The exceptionally strong association between delirium symptoms and cognitive impairment aligns closely with contemporary neurobiological models. Importantly, capturing delirium symptoms within a community-dwelling cohort, rather than in an acute hospital setting, highlights a state of early neurocognitive vulnerability rather than acute brain failure. These models posit that acute brain dysfunction, exemplified by delirium symptoms, is not merely a transient state but a pivotal pathophysiological event that could hypothetically unmask or accelerate underlying subclinical neurodegenerative processes, such as those seen in Alzheimer's disease or vascular cognitive impairment. Our findings suggest that in older adults with CHD, the presence of delirium symptoms may serve as a critical indicator of diminished cognitive reserve and a potential precursor to more severe IC impairment, thereby warranting heightened clinical vigilance and proactive cognitive screening. Moreover, anxiety and chronic constipation are not isolated complaints confined to psychological or gastrointestinal domains. Their co-occurrence appears to be linked to multisystem impairment in IC, spanning psychological wellbeing, cognition, locomotion, and vitality. This broad, cross-domain association suggests the possibility that these conditions may reflect deeper physiological dysregulation, such as dysfunction in the gut–brain axis and autonomic imbalance. Emerging evidence indicates that alterations in gut microbiota can influence central nervous system function through inflammatory pathways, vagal signaling, and neurotransmitter metabolism, such as serotonin (5-HT), thereby affecting mood and cognition (50–52). Conversely, chronic anxiety may further suppress gastrointestinal motility, creating a self-perpetuating cycle (53). Together, we hypothesize that this bidirectional brain–gut disruption might underlie a systemic frailty phenotype characterized by widespread capacity declines (54, 55).

An important methodological consideration of this study is the inherent conceptual overlap between several evaluated geriatric syndromes and IC domains. For instance, delirium symptoms are closely tied to cognition, just as dysphagia is to nutritional vitality. While the specific instruments used (e.g., EAT-10 vs. MNA-SF) do not share identical questionnaire items, they measure deeply interconnected clinical constructs. This overlap may partially explain the high strength of some observed associations. However, our sensitivity analysis—which excluded these overlapping combinations—demonstrated that the broader relationship between cumulative GS burden and IC impairment remains highly consistent.

Critically, many GS are frequently dismissed in clinical practice as minor, nonspecific, or benign symptoms, particularly in older adults who present primarily with cardiovascular disease. However, our findings suggest that these syndromes may instead serve as early sentinels of global physiological dysregulation rather than isolated complaints (16). From a clinical perspective, this highlights the need to integrate systematic GS screening into routine cardiovascular care for older adults. Rather than managing CHD in isolation, clinicians should proactively assess for high-yield syndromes such as delirium symptoms, anxiety, and chronic constipation, not only for their immediate symptom burden but also as indicators of impending multidomain IC impairment. While our cross-sectional study did not evaluate specific interventions, we propose that future clinical trials could explore whether targeted, syndrome-specific interventions, such as deprescribing anticholinergic medications to mitigate delirium symptoms (56), employing non-pharmacological strategies to address anxiety (57), or optimizing bowel regimens to alleviate constipation, might provide disproportionately broad benefits by simultaneously preserving multiple domains of IC. Integrating such assessments into standard cardiology workflows could facilitate the identification of high-risk individuals during a potentially modifiable window, enabling timely, multimodal interventions aimed at maintaining IC and fostering resilience in aging populations with chronic disease. This approach supports a paradigm shift toward integrated, function-oriented models of care, such as Comprehensive Geriatric Assessment (CGA), even within specialty settings like cardiology (58).

This study has several limitations. First, the cross-sectional design precludes causal inferences, necessitating future longitudinal studies. Second, reliance on self-reported data may introduce misclassification bias. Third, the lack of detailed clinical cardiovascular variables (e.g., myocardial infarction, heart failure, atrial fibrillation, revascularization) and precise CHD severity markers in this community survey may lead to residual confounding, despite our careful adjustment for chronic disease count and polypharmacy. Fourth, given the inherent conceptual overlap among specific geriatric syndromes, IC domains, and mental or psychological states (e.g., delirium risk and cognition), our adjusted models should be interpreted cautiously; these constructs are deeply interconnected, and statistical adjustment does not imply absolute clinical independence. Finally, these findings from community-dwelling older adults in China may not generalize to hospitalized populations. Nevertheless, the consistency of our results across multiple sensitivity and subgroup analyses reinforces the overall stability of our findings.

In conclusion, GS in community-dwelling older adults with self-reported physician-diagnosed CHD should not be viewed merely as comorbid conditions but as important indicators of multidomain capacity erosion. The progressive accumulation of these syndromes is strongly associated with intrinsic capacity impairment, reflecting a continuum that ranges from early capacity declines to advanced clinical frailty. Identifying high-risk syndromic patterns, particularly those involving delirium symptoms, anxiety, and chronic constipation, provides a strategic window for multimodal interventions aimed at preserving physiological reserves and fostering healthy aging.

## Data Availability

The original contributions presented in the study are included in the article/Supplementary material, further inquiries can be directed to the corresponding author/s.
