# Supplementary material for: Geriatric syndromes and intrinsic capacity in older adults with coronary heart disease: a cross-sectional study in China
Source: Front Public Health. 2026 Aug 14;14:1853890. doi: 10.3389/fpubh.2026.1853890 (PMC13521800; doi:10.3389/fpubh.2026.1853890)
Supplement: Supplementary file 1 [file Table_1.docx]

**Supplementary Table 1. Assessment instruments, raw assessment criteria, and score transformation procedures for IC domains**

| **IC Domain** | **Assessment Instrument** | **Raw Assessment Criteria** | **Transformed Score (0–20 scale)** | **References** |
| --- | --- | --- | --- | --- |
| **Cognition** | Chinese Mini-Mental State Examination (MMSE) | • MMSE ≥ 24 (Normal function) | • **20 points** | (24) |
|  |  | • MMSE 18–23 (Mild/Moderate impairment) | • **10 points** |  |
|  |  | • MMSE < 18 (Severe impairment) | • **0 points** |  |
| **Psychological Status** | 15-item Geriatric Depression Scale (GDS-15) | • GDS-15 ≤ 5 (Normal; no/minimal depressive symptoms) | • **20 points** | (25) |
|  |  | • GDS-15 6–9 (Mild depressive symptoms) | • **10 points** |  |
|  |  | • GDS-15 ≥ 10 (Severe depressive symptoms) | • **0 points** |  |
| **Vitality** | Mini-Nutritional Assessment Short-Form (MNA-SF) | • MNA-SF ≥ 12 (Normal; good nutritional status) | • **20 points** | (26) |
|  |  | • MNA-SF 8–11 (At risk of malnutrition) | • **10 points** |  |
|  |  | • MNA-SF ≤ 7 (Malnourished) | • **0 points** |  |
| **Sensory Function** | Self-reported vision and hearing impairment | • No impairment in either vision or hearing | • **20 points** |  |
|  |  | • Impairment in a single sensory organ (vision OR hearing) | • **10 points** |  |
|  |  | • Impairment in both sensory organs (vision AND hearing) | • **0 points** |  |
| **Locomotion** | Two items from SARC-F questionnaire (walking and stair-climbing difficulty) | • No difficulty in either task | • **20 points** | (27) |
|  |  | • Difficulty in only one task (some difficulty / great difficulty) | • **10 points** |  |
|  |  | • Difficulty in both tasks (some difficulty / great difficulty) | • **0 points** |  |

**Supplementary Table 2. Prevalence of specific GS in the overall sample and according to IC impairment status**

|  | **Overall** | **Without IC impairment** | **With IC impairment** | **p-value** |
| --- | --- | --- | --- | --- |
|  | **(n=5835)** | **(n=2679)** | **(n=3156)** |  |
| Polypharmacy |  |  |  |  |
| No | 5127 (87.9) | 2407 (89.8) | 2720 (86.2) | < 0.001 |
| Yes | 708 (12.1) | 272 (10.2) | 436 (13.8) |  |
| Poor sleep quality |  |  |  |  |
| No | 3541 (60.7) | 1884 (70.3) | 1657 (52.5) | < 0.001 |
| Yes | 2294 (39.3) | 795 (29.7) | 1499 (47.5) |  |
| Falls |  |  |  |  |
| No | 5073 (86.9) | 2484 (92.7) | 2589 (82.0) | < 0.001 |
| Yes | 762 (13.1) | 195 (7.3) | 567 (18.0) |  |
| High risk of osteoporosis |  |  |  |  |
| No | 4375 (75.0) | 2265 (84.5) | 2110 (66.9) | < 0.001 |
| Yes | 1460 (25.0) | 414 (15.5) | 1046 (33.1) |  |
| Anxiety |  |  |  |  |
| No | 5251 (90.0) | 2554 (95.3) | 2697 (85.5) | < 0.001 |
| Yes | 584 (10.0) | 125 (4.7) | 459 (14.5) |  |
| Chronic pain |  |  |  |  |
| No | 4187 (71.8) | 2108 (78.7) | 2079 (65.9) | < 0.001 |
| Yes | 1648 (28.2) | 571 (21.3) | 1077 (34.1) |  |
| Dysphagia |  |  |  |  |
| No | 5514 (94.5) | 2621 (97.8) | 2893 (91.7) | < 0.001 |
| Yes | 321 (5.5) | 58 (2.2) | 263 (8.3) |  |
| Delirium symptoms |  |  |  |  |
| No | 5627 (96.4) | 2656 (99.1) | 2971 (94.1) | < 0.001 |
| Yes | 208 (3.6) | 23 (0.9) | 185 (5.9) |  |
| Chronic constipation |  |  |  |  |
| No | 5297 (90.8) | 2511 (93.7) | 2786 (88.3) | < 0.001 |
| Yes | 538 (9.2) | 168 (6.3) | 370 (11.7) |  |
| No. of GS |  |  |  |  |
| 0 | 1776 (30.4) | 1107 (41.3) | 669 (21.2) | < 0.001 |
| 1 | 1743 (29.9) | 873 (32.6) | 870 (27.6) |  |
| 2 | 1128 (19.3) | 459 (17.1) | 669 (21.2) |  |
| 3 | 624 (10.7) | 154 (5.7) | 470 (14.9) |  |
| ≥4 | 564 (9.7) | 86 (3.2) | 478 (15.1) |  |
| Data are presented as n (%). The χ² test was conducted for categorical variables. GS = Geriatric Syndrome, IC = Intrinsic Capacity. | | | | |

**Supplementary Table 3. Independent associations of specific GS pairs with IC impairment after adjusting for total concurrent syndrome burden.**

|  | **IC impairment** |
| --- | --- |
|  | PR (95% CI, p-value) |
| **Comorbid GS Combination** |  |
| **Anxiety + Chronic constipation** | 1.29 (1.04-1.61, p = 0.023) |
| **Delirium symptoms + Anxiety** | 1.19 (0.94-1.52, p = 0.154) |
| **Delirium symptoms + Chronic constipation** | 1.15 (0.82-1.62, p = 0.423) |
| **Poor sleep quality + Delirium symptoms** | 1.24 (1.03-1.49, p = 0.021) |
| The model was adjusted for age, sex, ethnicity, national region, residence, education level, marital status, occupation, dwelling status, alcohol consumption, smoking status, number of chronic diseases, and the total geriatric syndrome burden (i.e., the count of concurrent syndromes excluding the specific pair being evaluated).The adjusted PRs were estimated using multivariable modified Poisson regression models. PR = Prevalence Ratio, CI=Confidence Interval, IC=Intrinsic Capacity, GS=Geriatric Syndrome. | |

**Supplementary Table 4. Sensitivity analysis for the association between GS burden and IC impairment, additionally adjusted for frailty and disability**

|  | **IC impairment** | **Locomotion** | **Cognition** | **Sensory** | **Vitality** | **Psychological** |  |
| --- | --- | --- | --- | --- | --- | --- | --- |
|  | PR (95% CI, p-value) | PR (95% CI, p-value) | PR (95% CI, p-value) | PR (95% CI, p-value) | PR (95% CI, p-value) | PR (95% CI, p-value) |  |
| **No. of GS** |  |  |  |  |  |  |  |
| **0** | 1.00 (reference) | 1.00 (reference) | 1.00 (reference) | 1.00 (reference) | 1.00 (reference) | 1.00 (reference) |  |
| 1 | 1.25 (1.13-1.39, p < 0.001) | 1.43 (1.09-1.88, p = 0.010) | 1.56 (1.23-1.97, p < 0.001) | 1.27 (1.11-1.44, p < 0.001) | 1.30 (1.03-1.64, p = 0.029) | 1.43 (1.09-1.87, p = 0.009) |  |
| 2 | 1.41 (1.26-1.58, p < 0.001) | 2.04 (1.55-2.69, p < 0.001) | 1.64 (1.27-2.12, p < 0.001) | 1.43 (1.24-1.65, p < 0.001) | 1.27 (0.98-1.64, p = 0.065) | 2.09 (1.59-2.75, p < 0.001) |  |
| 3 | 1.68 (1.48-1.90, p < 0.001) | 2.81 (2.11-3.73, p < 0.001) | 2.32 (1.77-3.04, p < 0.001) | 1.90 (1.63-2.22, p < 0.001) | 1.43 (1.07-1.90, p = 0.015) | 2.85 (2.13-3.81, p < 0.001) |  |
| ≥4 | 1.73 (1.52-1.97, p < 0.001) | 3.30 (2.49-4.38, p < 0.001) | 3.11 (2.38-4.06, p < 0.001) | 2.06 (1.75-2.42, p < 0.001) | 1.52 (1.14-2.03, p = 0.004) | 5.10 (3.87-6.74, p < 0.001) |  |
| **Comorbidity** |  |  |  |  |  |  |  |
| **Polypharmacy** |  |  |  |  |  |  |  |
| No | 1.00 (reference) | 1.00 (reference) | 1.00 (reference) | 1.00 (reference) | 1.00 (reference) | 1.00 (reference) |  |
| Yes | 1.03 (0.93-1.14, p = 0.613) | 1.32 (1.09-1.59, p = 0.004) | 1.22 (1.00-1.49, p = 0.048) | 1.06 (0.93-1.21, p = 0.366) | 0.89 (0.70-1.13, p = 0.331) | 0.95 (0.76-1.19, p = 0.660) |  |
| **Poor sleep quality** |  |  |  |  |  |  |  |
| No | 1.00 (reference) | 1.00 (reference) | 1.00 (reference) | 1.00 (reference) | 1.00 (reference) | 1.00 (reference) |  |
| Yes | 1.22 (1.14-1.32, p < 0.001) | 1.34 (1.15-1.57, p < 0.001) | 1.41 (1.21-1.64, p < 0.001) | 1.32 (1.20-1.45, p < 0.001) | 1.11 (0.94-1.31, p = 0.222) | 1.61 (1.37-1.90, p < 0.001) |  |
| **Falls** |  |  |  |  |  |  |  |
| No | 1.00 (reference) | 1.00 (reference) | 1.00 (reference) | 1.00 (reference) | 1.00 (reference) | 1.00 (reference) |  |
| Yes | 1.22 (1.11-1.34, p < 0.001) | 1.96 (1.67-2.31, p < 0.001) | 1.41 (1.17-1.69, p < 0.001) | 1.26 (1.12-1.41, p < 0.001) | 1.04 (0.84-1.28, p = 0.714) | 1.81 (1.52-2.17, p < 0.001) |  |
| **High risk of osteoporosis** |  |  |  |  |  |  |  |
| No | 1.00 (reference) | 1.00 (reference) | 1.00 (reference) | 1.00 (reference) | 1.00 (reference) | 1.00 (reference) |  |
| Yes | 1.29 (1.19-1.39, p < 0.001) | 1.54 (1.32-1.81, p < 0.001) | 1.36 (1.16-1.60, p < 0.001) | 1.39 (1.26-1.54, p < 0.001) | 1.30 (1.09-1.55, p=0.004) | 2.09 (1.78-2.47, p < 0.001) |  |
| **Anxiety** |  |  |  |  |  |  |  |
| No | 1.00 (reference) | 1.00 (reference) | 1.00 (reference) | 1.00 (reference) | 1.00 (reference) | 1.00 (reference) |  |
| Yes | 1.26 (1.14-1.40, p < 0.001) | 1.34 (1.11-1.62, p = 0.002) | 1.67 (1.38-2.02, p < 0.001) | 1.37 (1.21-1.56, p < 0.001) | 1.26 (1.02-1.57, p = 0.034) | 3.13 (2.63-3.72, p < 0.001) |  |
| **Chronic pain** |  |  |  |  |  |  |  |
| No | 1.00 (reference) | 1.00 (reference) | 1.00 (reference) | 1.00 (reference) | 1.00 (reference) | 1.00 (reference) |  |
| Yes | 1.16 (1.07-1.25, p < 0.001) | 1.47 (1.26-1.72, p < 0.001) | 1.26 (1.07-1.47, p = 0.004) | 1.28 (1.16-1.41, p < 0.001) | 0.94 (0.79-1.13, p=0.525) | 1.25 (1.06-1.48, p = 0.008) |  |
| **Dysphagia** |  |  |  |  |  |  |  |
| No | 1.00 (reference) | 1.00 (reference) | 1.00 (reference) | 1.00 (reference) | 1.00 (reference) | 1.00 (reference) |  |
| Yes | 1.26 (1.11-1.43, p < 0.001) | 1.72 (1.39-2.12, p < 0.001) | 1.70 (1.35-2.14, p < 0.001) | 1.42 (1.22-1.66, p < 0.001) | 1.63 (1.28-2.08, p < 0.001) | 2.30 (1.85-2.86, p < 0.001) |  |
| **Delirium symptoms** |  |  |  |  |  |  |  |
| No | 1.00 (reference) | 1.00 (reference) | 1.00 (reference) | 1.00 (reference) | 1.00 (reference) | 1.00 (reference) |  |
| Yes | 1.29 (1.11-1.51, p = 0.001) | 1.37 (1.06-1.77, p = 0.017) | 3.34 (2.69-4.14, p < 0.001) | 1.41 (1.17-1.70, p < 0.001) | 1.32 (0.97-1.80, p = 0.081) | 2.91 (2.30-3.69, p < 0.001) |  |
| **Chronic constipation** |  |  |  |  |  |  |  |
| No | 1.00 (reference) | 1.00 (reference) | 1.00 (reference) | 1.00 (reference) | 1.00 (reference) | 1.00 (reference) |  |
| Yes | 1.18 (1.06-1.32, p = 0.003) | 1.42 (1.16-1.73, p < 0.001) | 1.39 (1.13-1.72, p = 0.002) | 1.16 (1.01-1.33, p = 0.042) | 1.38 (1.10-1.72, p = 0.005) | 1.49 (1.21-1.85, p < 0.001) |  |
| The model was adjusted for age, sex, ethnicity, national region, residence, education level, marital status, occupation, dwelling status, alcohol consumption, smoking status, and number of chronic diseases. The adjusted PRs were estimated using multivariable modified Poisson regression models. PR = Prevalence Ratio, CI = Confidence Interval, IC = Intrinsic Capacity, GS = Geriatric Syndrome. | | | | | | |  |

**Supplementary Table 5. Sensitivity analysis for the association between cumulative GS burden and IC impairment, after excluding patients with severe cognitive impairment and malnutrition.**

|  | **IC impairment** | **Locomotion** | **Cognition** | **Sensory** | **Vitality** | **Psychological** |  |
| --- | --- | --- | --- | --- | --- | --- | --- |
|  | PR (95% CI, p-value) | PR (95% CI, p-value) | PR (95% CI, p-value) | PR (95% CI, p-value) | PR (95% CI, p-value) | PR (95% CI, p-value) |  |
| **No. of GS** |  |  |  |  |  |  |  |
| **0** | 1.00 (reference) | 1.00 (reference) | 1.00 (reference) | 1.00 (reference) | 1.00 (reference) | 1.00 (reference) |  |
| 1 | 1.28 (1.15-1.42, p < 0.001) | 1.67 (1.26-2.20, p < 0.001) | 1.55 (1.21-1.99, p < 0.001) | 1.29 (1.13-1.48, p < 0.001) | 1.43 (1.13-1.81, p = 0.003) | 1.49 (1.13-1.94, p = 0.004) |  |
| 2 | 1.49 (1.33-1.67, p < 0.001) | 2.86 (2.17-3.77, p < 0.001) | 1.62 (1.24-2.13, p < 0.001) | 1.50 (1.30-1.73, p < 0.001) | 1.52 (1.17-1.97, p = 0.002) | 2.32 (1.76-3.04, p < 0.001) |  |
| 3 | 1.86 (1.64-2.10, p < 0.001) | 4.56 (3.43-6.06, p < 0.001) | 2.52 (1.89-3.35, p < 0.001) | 2.01 (1.72-2.35, p < 0.001) | 1.91 (1.43-2.56, p < 0.001) | 3.38 (2.53-4.51, p < 0.001) |  |
| ≥4 | 2.01 (1.76-2.29, p < 0.001) | 6.20 (4.68-8.22, p < 0.001) | 3.65 (2.77-4.81, p < 0.001) | 2.20 (1.87-2.59, p < 0.001) | 2.33 (1.74-3.12,p < 0.001) | 6.30 (4.79-8.28, p < 0.001) |  |
| The sensitivity analysis excluded patients with severe cognitive impairment (defined as MMSE < 18) and severe malnutrition (defined as MNA-SF ≤ 7). The model was adjusted for age, sex, ethnicity, national region, residence, education level, marital status, occupation, dwelling status, alcohol consumption, smoking status, and number of chronic diseases. The adjusted PRs were estimated using multivariable modified Poisson regression models. PR = Prevalence Ratio, CI = Confidence Interval, IC = Intrinsic Capacity, GS = Geriatric Syndrome. | | | | | | |  |

**Supplementary Table 6. Association between cumulative GS burden and impairment across five IC domains in community-dwelling older adults with self-reported physician-diagnosed CHD.**

|  | **Locomotion** | **Cognition** | **Sensory** | **Vitality** | **Psychological** |
| --- | --- | --- | --- | --- | --- |
|  | PR (95% CI, p-value) | PR (95% CI, p-value) | PR (95% CI, p-value) | PR (95% CI, p-value) | PR (95% CI, p-value) |
| **No. of GS** |  |  |  |  |  |
| **0** | 1.00 (reference) | 1.00 (reference) | 1.00 (reference) | 1.00 (reference) | 1.00 (reference) |
| **1** | 1.70 (1.37-2.12, p < 0.001) | 1.50 (1.20-1.89, p < 0.001) | 1.29 (1.13-1.47, p < 0.001) | 1.44 (1.14-1.80, p = 0.002) | 1.50 (1.17-1.93, p = 0.002) |
| **2** | 2.64 (2.09-3.33, p < 0.001) | 1.67 (1.31-2.14, p < 0.001) | 1.48 (1.29-1.71, p < 0.001) | 1.57 (1.22-2.01, p < 0.001) | 2.33 (1.81-3.01, p < 0.001) |
| **3** | 3.78 (2.90-4.93, p < 0.001) | 2.46 (1.90-3.20, p < 0.001) | 2.01 (1.73-2.35, p < 0.001) | 1.81 (1.36-2.41, p < 0.001) | 3.13 (2.36-4.14, p < 0.001) |
| **≥4** | 6.36 (4.83-8.36, p < 0.001) | 3.35 (2.60-4.33, p < 0.001) | 2.24 (1.92-2.62, p < 0.001) | 2.49 (1.88-3.31, p < 0.001) | 5.72 (4.37-7.50, p < 0.001) |
| The model was adjusted for age, sex, ethnicity, national region, residence, education level, marital status, occupation, dwelling status, alcohol consumption, smoking status, and number of chronic diseases. The adjusted PRs were estimated using multivariable modified Poisson regression models. PR = Prevalence Ratio, CI = Confidence Interval, IC = Intrinsic Capacity, GS = Geriatric Syndrome. | | | | | |
